# Molecular Investigation of Pediatric Portuguese Patients with Sensorineural Hearing Loss

**DOI:** 10.4061/2011/587602

**Published:** 2011-09-25

**Authors:** Célia Nogueira, Miguel Coutinho, Cristina Pereira, Alessandra Tessa, Filippo M. Santorelli, Laura Vilarinho

**Affiliations:** ^1^Genetics Department, Medical Genetics Center, National Institute of Health (INSA), Praça Pedro Nunes 88, 4099-028 Porto, Portugal; ^2^Otorhinolaryngology Department, Children's Hospital Maria Pia, Rua da Boavista 827, 4050-111 Porto, Portugal; ^3^Molecular Medicine and Neurogenetics, IRCCS Fondazione Stella Maris, Viale del Tirreno 331, 56128 Pisa, Italy

## Abstract

The understanding of the molecular genetics in sensorineural hearing loss (SNHL) has advanced rapidly during the last decade, but the molecular etiology of hearing impairment in the Portuguese population has not been investigated thoroughly. To provide appropriate genetic testing and counseling to families, we analyzed the whole mitochondrial genome in 95 unrelated children with SNHL (53 nonsyndromic and 42 syndromic) and searched for variations in two frequent genes, *GJB2* and *GJB6*, in the non-syndromic patients. Mutations in mtDNA were detected in 4.2% of the cases, including a hitherto undescribed change in the mtDNA-tRNA^Trp^ gene (namely, m.5558A>G). We also identified mono- or biallelic *GJB2* mutations in 20 of 53 non-syndromic cases and also detected two novel mutations (p.P70R and p.R127QfsX84). Our data further reinforce the notion that genetic heterogeneity is paramount in children with SNHL.

## 1. Introduction

Sensorineural hearing loss (SNHL) is one of the most common disabilities in human, and genetics is an important aspect in research and clinical practice for SNHL. One in 1000 children is born with bilateral SNHL, and 50–70% of them have monogenic causes for their deafness [1]. In addition, 10% of the people over 65 years have SNHL that limits considerably speech communication. Although the etiology is polygenic in most of the cases, with different contribution of ageing and environment, discovering monogenic causes has important clinical implications in terms of better counseling and management.

Hereditary hearing loss can be classified into syndromic and nonsyndromic depending on the associated features. Whilst over 400 genetic syndromes have been described in association with mono- or bilateral deafness, syndromic conditions account for about 30% of hereditary congenital hearing loss, whereas the relative contribution to all deaf people is much higher (>70%) for nonsyndromic subtypes [1].

Nonsyndromic sensorineural hearing loss (NSSNHL) is predominantly inherited in an autosomal recessive patterns (DFNB loci) (80%) but can be also autosomal dominantly (DFNA) (15–20%), X-linked (DFN) (2-3%), or maternally (1%) transmitted. A polygenic or multifactorial pattern of inheritance should be postulated for late onset cases of hearing impairment [2, 3]. To date, 134 deafness loci (77 DFNB and 57 DFNA) have been reported, with more than 40 genes cloned [4]. Further heterogeneity is, however, expected to emerge.

Mutations in the *GJB2* and *GJB6* genes on the DFNB1 locus at chromosome 13q11-q12 are responsible for up to 50% of autosomal recessive (AR) NSSNHL [5, 6]. *GJB2* and *GJB6* encode gap junction proteins connexin 26 (Cx26) and connexin 30 (Cx30), respectively, which are expressed in the cochlea where they colocalize, form heteromeric gap junctions [7], and play a role in cochlear homeostasis [8]. The list of allelic variants in *GJB2* is wide with more than 100 variants being detected, mainly in congenital AR deafness, but also in dominant forms. The deletion of a single guanine (c.35delG) individually account for up to 50% of cases of NSSNHL among populations in Europe, North America, and Asia [9, 10]. The common 342-Kb genomic deletion in* GJB6 *(termed GJB6-D13S1830) occurs in up to 20% of the hearing-impaired American population and may account for ~10% of all DFNB1 alleles with an extremely wide range based on ethnic origin, oftentimes in dysgenic association with the c.35delG/*GJB2* variant [11, 12]. 

Maternally inherited hearing loss account for approximately 1% of cases, but accurate diagnosis is important because of its unique implications for affected individuals and their family members. Hearing loss may be an early manifestation of a more complex mitochondrial disorder such as the Kearns-Sayre (KSS) [13–15], the MERRF (myoclonic epilepsy with ragged-red fibers) [16, 17], or the MELAS (mitochondrial encephalomyopathy, lactic acidosis, and stroke-like episodes) [18, 19] syndromes. In addition, SNHL can be the main, or sole, presenting feature of a mtDNA defect. In particular, mutations in the mtDNA-encoded 12S rRNA and tRNA^Ser(UCN)^ genes are frequent. The m.1555A>G mutation in 12S rRNA has a worldwide distribution, and it is localized in a highly conserved region which is involved in decoding small ribosomal subunit [20]. The new G–C pair in 12S rRNA created by the A>G transition facilitates the binding of aminoglycosides antibiotics such as gentamycin and streptomycin, causing aminoglycoside-induced NSSNHL or worsening hearing loss in individuals harboring this mutation. The prevalence of the m.1555A>G mutation has been shown to be between 20–30% in deaf individuals in Spain and Asia of which 15% of them had the history of aminoglycoside ototoxicity [21]. Additional mutations in 12S rRNA (m.961delT, m.961insC(n), m.1005T>C, m.1095T>C, and m.1494 C>T) have been associated with impaired hearing [22–25], although their pathogenic significance remains controversial. All this information indicates that the mitochondrial 12S rRNA gene is a hotspot for mutations causing nonsyndromic hearing loss as well as increased sensitivity to aminoglycoside ototoxicity [26]. Mutations in the tRNA^Ser(UCN)^ gene are additional genetic hotspot in Caucasian and Asian pedigrees with nonsyndromic and aminoglycoside-induced hearing loss. In the vast majority of patients, mtDNA mutations are nearly homoplasmic, indicating the requirement of a high mutation load to exert cochlear dysfunction [27].

We performed a comprehensive analysis of *GJB2* and *GJB6* in 53 non-syndromic patients and scanned the whole mitochondrial genome for mutations in a cohort of 95 paediatric Portuguese SNHL patients recruited in a hearing clinic to investigate the relative contribution of common genetic etiologies to their disorder, provide effective assessment of genetic risk, and to better counsel their relatives.

## 2. Patients and Methods

### 2.1. Subjects

Ninety-five Portuguese SNHL children from unrelated families were included in this study. A diagnosis of hearing deficit was performed in a Hearing Clinic according to international guidelines [28]. Fifty-three cases (56%) showed a non-syndromic hearing loss, profound in 80% and mild to moderate in 20%, and 42 (44%) patients had a syndromic disorder. Frequent accompanying manifestations involved the nervous system, eye, external ear, and musculoskeletal system. Parents were interviewed with regard to age of onset, family history, mother's health during pregnancy, and patient's past clinical history, including infection, possible head or brain injury, and the use of aminoglycoside antibiotics. Written informed consent was obtained from all patients or their parents prior to blood sampling and all clinical and molecular studies accomplished with the ethical issues of Declaration of Helsinki.

### 2.2. DNA Extraction

Total genomic DNA was extracted from peripheral blood (B) or skeletal muscle (M) using commercially available procedures which employed the *EZ1 DNA Blood 350 *μ*L Kit (QIAGEN)* and *Puregene Tissue kit* (Gentra) kits, respectively.

### 2.3. Mutational Analyses

We performed polymerase chain reaction (PCR) amplifications and direct gene sequencing of the coding exon and flanking regions of *GJB2* in 53 non-syndromic patients, as described elsewhere [5]. The common* GJB6*-D13S1830 deletion [29] was searched in patients harbouring a heterozygous sequence variation in *GJB2*. All 95 patients were screened for mutations in the entire mtDNA by using a commercially available kit, mitoSEQr Resequencing System for the Human Mitochondrial Genome (Applied Biosystems, Foster City, Calif), according to the procedure recommended by the manufacturer.

For the description of the mutations, we used the latest conventions of the Human Genome Variation Society nomenclature. Synonymous, missense, and splice site variations were systematically evaluated for modifications of exonic splicing enhancers (Polyphen analysis, http://genetics.bwh.harvard.edu/pph/; ESEfinder, http://rulai.cshl.edu/cgi-bin/tools/ESE/esefinder.cgi) or consensus splicing sequences in order to determine the splice site score (http://www.cbs.dtu.dk/services/NetGene2/ and http://www.fruitfly.org/seq_tools/splice.html). Multiple alignments with *GJB2* orthologs were performed using ClustalW (http://www.ebi.ac.uk/Tools/msa/clustalw2/) to evaluate the degree of conservation of missense variants. MtDNA mutations and polymorphisms were searched in MITOMAP data base (http://mitomap.org/MITOMAP). 

## 3. Results

Table 1 summarizes molecular data detected in children with SNHL. Sequence analysis of *GJB2 *indicated that eight patients carried two mutated alleles with already described pathogenic mutations and one presented the p.M34T mutation in heterozogosity, which has been reported to cause AD-NSSNHL [30]. The frequent c.35delG was homozygous in four children and was detected on a single allele in eight patients, three of whom harbored a wild-type sequence on the other allele and two children harbored the heterozygous common (GJB6-D13S1830) deletion. Two additional patients presented novel variations in *GJB2*: a homozygous p.R127QfsX84 mutation was found in one case whereas another patient presented the p.P70R on a single allele. Four patients presented with heteroplasmic (two individuals) or homoplasmic (two cases) mutations in mtDNA, including the hitherto unreported m.5558A>G in the mtDNA-encoded tRNA^Trp^ gene. 

Collectively, about 17% (16/95) of the Portuguese children analyzed in the present study were molecularly characterized, whereas a single variant was identified in four patients. The c.35delG accounted for 40% (16/40) of all mutant alleles, and up to half of mutations occurring in connexin-related genes. Additionally, 4.2% of studied patients (4/95) presented mutations in mtDNA, two of them (m.5558A>G and m.7445A>G) were identified in non-syndromic patients and the remaining mutations (m.1555A>G and m.3243A>G) in syndromic cases.

## 4. Discussion

Despite increasing evidence of mutations associated with SNHL, there have been thus far no studies reporting on the relative frequency of mutations in the hearing-impaired Portuguese pediatric population. Although limited to a single center and to a relatively small number of cases, our study demonstrated that screening of two common etiologies (*GJB2* and* GJB6*) can characterize one in five patients with NSSNHL of unknown etiology. The relative frequency of the recurrent c.35delG mutation—64% of all mutated *GJB2* alleles—is in agreement with data reported by others [9, 10], as well as, the whole mtDNA in all SNHL patients.

In the present investigation, mutations in the *GJB2* gene were very frequent among NSSNHL patients, accounted for 23.6% (25/106) of mutated alleles and appear particularly frequent in South European patients [9, 10]. Among the DFNB-causing *GJB2* gene mutations reported so far, the c.35delG mutation accounts for most of mutant alleles (60–85%) in Caucasians, from which 10–50% was present in only one allele. Our data confirms the presence of the common* GJB6*-D13S1830 deletion in two patients, showing a digenic inheritance in keeping with the severity of their hearing deficit.

We also identified two possibly pathogenic novel variants in *GJB2*. The new p.P70R that substitutes a proline (iminoacid) at codon 70 to an arginine (basic aminoacid) was predicted to be probably damaging by PolyPhen (The score is 2.108), and arginine was not tolerated when analyzed with the prediction software SIFT. Whether the mutation results in NSSNHL inherited in a dominant fashion remains a possibility. Conversely, the novel p.R127QfsX84 predicts a frameshift with a shorter, prematurely truncated connexin-26.

We also detected four mutations in mtDNA, corresponding to 4.2% of the all cohort. The m.1555A>G mutation has been reported in families whose members presented with aminoglycoside-induced deafness as the sole pathologic feature, but it may cause hearing loss even without aminoglycoside exposure, as in our case, which represents ~1% of this cohort. In our study, this mutation was found in a patient with ataxia and failure to thrive. The m.3243A>G mutation determines not only nonsyndromic, but also syndromic SNHL, such as in MELAS and MIDD (maternally inherited diabetes and deafness) [1]. The case identified in the present work was a 12-year-old boy diagnosed at the age of 8 years with syndromic SNHL; he developed hypertrophic cardiomyopathy and generalized muscle atrophy four years later. The m.7445A>G mutation was detected in a 13-year-old boy who presented a history of moderate progressive hearing loss. This mutation is believed to have led to failure in the processing of the L-strand RNA precursor, thereby reducing the steady-state levels of tRNA^Ser(UCN)^ and ND6 mRNA [27].

**Figure 1:**
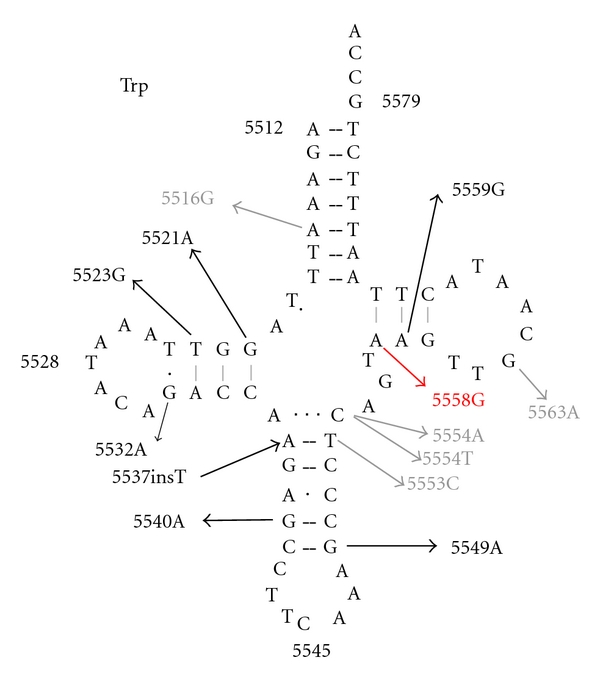
Schematic secondary structure of the wild-type human mitochondrial tRNA^Trp^ gene showing the reported variants found in this gene. Black stands for pathogenic mutations, gray stands for polymorphic mutations, and red stands for the novel mutation described in our study.

We also found a novel mutation (m.5558A>G) in the tRNA^Trp^ in a 10-year-old boy with NSSNHL. The mutation affects the conserved A49 nucleotide in the T-stem of the tRNA (Figure 1) leading to a possible disruption of its secondary structure after the loss of the T-stem. This modification could also affect the tertiary structure of the tRNA^Trp^ and has probably a consequence on the aminoacylation. Another mutation (m.5559A>G) has been reported in the T-stem of the tRNA^Trp^ [35], and two additional tRNA^Trp^ mutations (namely, m.5540A>G and m.5568A>G) have been associated with SNHL [36, 37].

## 5. Conclusions

Although the contribution of less common genes remains to be determined, our results suggest that analysis of the *GJB2 *gene may have clinical implications in the diagnosis of deaf Portuguese children. Also, it would make feasible early rehabilitation and prevention in affected families. The relatively higher incidence of mtDNA mutation also suggests that screening for variations in the mitochondrial genome should always be considered unless mitochondrial inheritance can be excluded for certain. The molecular diagnosis will permit more accurate genetic counseling for family members, monitor possible multisystem complications, and avoid usage of aminoglycosides if infections occur.

## Figures and Tables

**Table 1:** Genotype of patients with mutations in nuclear genes (GJB2 and GJB6) and in mitochondrial genome.

Number of patients	*GJB2*	Reference	*GJB6*	Reference	mtDNA	Reference
4	c.35delG/c.35delG	[9]	—		—	
1	p.W24X/p.W24X	[30]	—		—	
1	c.35delG/p.E47X	[9, 31]	—		—	
1	c.35delG/p.L76P	[9, 32]	—		—	
1	c.35delG/p.V95M	[9, 33]	—		—	
1	p.M34T/wt	[30]	—		—	
1	p.R127QfsX84/p.R127QfsX84	This study	—		—	
3	c.35delG/wt	[9]	—		—	
1	p.P70R/wt	This study	—		—	
2	c.35delG	[9]	del(GJB6-D13S1830)	[29]	—	
1	—		—		m.7445A>G	[34]
1	—		—		m.1555A>G	[20]
1	—		—		m.3243 A>G	[19]
1	—		—		m.5558 A>G	This study
